# The roles of primary care doctors in the COVID-19 pandemic: consistency and influencing factors of doctor's perception and actions and nominal definitions

**DOI:** 10.1186/s12913-022-08487-0

**Published:** 2022-09-09

**Authors:** Chenbin Yang, Jiana Yin, Jiongjiong Liu, Jinying Liu, Qin Chen, Hui Yang, Yunchao Ni, Bingcan Li, Yanmei Li, Jin Lin, Ziwei Zhou, Zhangping Li

**Affiliations:** 1 Department of Emergency, The Quzhou Affiliated Hospital of Wenzhou Medical University, Quzhou People’s Hospital, Quzhou, 324000 China; 2 Department of Emergency, The first affiliated Hospital of Wenzhou Medical University, Wenzhou, 325005 China; 3 School of Primary Care and Allied Health, Faculty of Medicine, Nursing and Health Sciences, Monash University, VIC, 3168 Australia; 4 Department of General practice, The People’s Hospital of Yueqing, Wenzhou, 325600 China; 5 Wenzhou Medical University, Wenzhou, 325005 China; 6 Wenzhou Medical University Renji College, Wenzhou, 325005 China

**Keywords:** Primary care doctors, Role perception, COVID-19, Questionnaire

## Abstract

**Background:**

At the end of 2019, the Coronavirus Disease 2019 (COVID-19) pandemic broke out. As front-line health professionals, primary care doctors play a significant role in screening SARS-CoV-2 infection and transferring suspected cases. However, the performance of primary care doctors is influenced by their knowledge and role perception. A web-based cross-sectional survey was conducted to assess the consistency and influencing factors of primary care doctor's role perception and expert advice in the guidelines (regulatory definition).

**Methods:**

We designed the questionnaire using “Wenjuanxing” platform, distributed and collected the questionnaire through WeChat social platform, and surveyed 1758 primary care doctors from 11 community health service stations, community health service centers and primary hospitals in Zhejiang Province, China. After the questionnaire was collected, descriptive statistics were made on the characteristics of participants, and univariate analysis and multivariate analysis were used to determine the relevant factors affecting their role cognition.

**Results:**

In the reporting and referral suspected cases and patients receiving treatment, most participants’ cognition of their roles were consistent with the requirements of guidelines. However, 49.54% and 61.43% of participant doctors were not in line with the government guidelines for diagnosing and classifying COVID-19 and treating suspected cases, respectively. Having a middle or senior professional title and participating in front-line COVID-19 prevention and control work is beneficial to the accurate role perception of diagnosis and classification of COVID-19, the reporting and transfer of suspected cases, and the treatment of suspected cases.

**Conclusions:**

Primary care doctors’ role perceptions in the COVID-19 pandemic are not always consistent with government guidelines in some aspects, such as transferring and diagnosing suspected cases. Therefore, it is essential to guide primary care doctors in performing their duties, especially those with lower professional titles.

**Supplementary Information:**

The online version contains supplementary material available at 10.1186/s12913-022-08487-0.

## Background

Coronavirus Disease 2019 (COVID-19) have been reported in almost all countries, giving rise to a worldwide pandemic [1] and becoming a current global health threat and a public health event of international concern [2]. In the early days of the outbreak, the total number of cases and deaths exceeded SARS [3]. Subsequently, the virus spread exponentially and spread to the whole world. As of March 4, 2022, 442,506,369 confirmed cases of 2019-nCoV were reported, and a total of 5,982,506 deaths were recorded (https://coronavirus.jhu.edu/map.html). In facing the COVID-19 pandemic, there is no effective cure at present. But early identification of symptoms and timely adoption of effective preventive measures will help patients recover as soon as possible and prevent virus transmission [4], revealing the importance of primary health care. Primary care doctors play a significant role in the prevention and management activities.

In fact, in many countries, primary care doctors are part of the surveillance system for infectious diseases such as influenza [5–9]. They are the gatekeepers of the health care system [5, 10]. It is reported that primary care doctors greatly influence the vaccination of the H1N1 vaccine during the H1N1 pandemic [11, 12]. The role of the vaccine is mainly to stimulate the body to produce specific antibodies against the virus by activating the human immune system. After receiving the COVID-19 vaccine, the body may be more resistant to the virus, resulting in an immune effect. Therefore, it is recommended to actively vaccinate in the absence of special contraindications to promote the normal production of antibodies. However, for new pandemic vaccines, vaccine hesitation and refusal may be the main obstacles to vaccination. Primary health care doctors play a key role in conducting vaccine education and popularizing the safety and effectiveness of vaccines. They provide advice on vaccines to patients and publicize that vaccination plans and policies can effectively control pandemics. Earlier studies have shown that primary health care is related to a more equitable distribution of health worldwide [13]. A recent study in Georgia pointed to the potential importance of vital primary health care in reducing COVID-19 mortality [14].

As part of medical reform efforts in China, primary health care aims to provide citizens with universal and fair high-quality medical services [15]. Since the first outbreak of COVID-19 in Wuhan, Hubei Province, 970,000 primary healthcare institutions have participated in screening for SARS-CoV-2 infection and managing suspected cases for COVID-19 prevention and control, involving more than 4 million primary healthcare doctors [16]. These primary health care institutions include community health service stations, township health centers, community health service stations, and village clinics [17]. Primary health care institutions in China are mainly responsible for COVID-19 screening and referral, monitoring, education, and publicity [18, 19]. Evidence of the impact of primary care doctors on health promotion has accumulated, proving that they play a vital role in epidemic management, and well-integrated primary health care and public health system is essential for a unified response [20]. The primary medical and health institutions of China have limited epidemic prevention capabilities. Thus, work guidelines that conform to the characteristics and actual conditions of the grass-roots front-line are required. The China Primary Respiratory Disease Prevention and Control Alliance took the lead and invited experts in respiratory, general practice, public health and other related fields to jointly formulate the ‘Expert recommendations for the prevention and control of novel coronavirus infections in primary care’. Detailed guidance was given in the following terms: 1) pre-examination and triage form based on epidemiological history and clinical manifestation for screening SARS-CoV-2; 2) the procedures for quarantine and management of suspected COVID-19 cases; 3) Home follow-up of discharged patients with SARS-CoV-2 infection, centralized or home isolation of close contacts, and management procedures; 4)Transfer procedures for suspected COVID-19 cases in primary healthcare institutions; 5) Community education on scientific prevention and control of COVID-19 infection. However, it is unclear whether primary care doctors are well aware of their role in optimizing the prevention and control of disease in conjunction with other governmental departments and institutions.

In this study, we investigated primary care doctors in Zhejiang Province to determine the consistency of their role perception and expert advice, aiming to provide a basis for targeted education and amend their role perception in significant public health incidents.

## Methods

### Participant enrollment and experimental design

The participants were primary care doctors randomly selected from 11 cities of Zhejiang Province through WeChat social platform, and 1,758 valid questionnaires were collected. The sample size was determined by the formula proposed by Tabachnick and Fidell [21]. The informed consent of all participants was obtained before the interview, and this procedure was approved by the Ethics Committee in Clinical Research (ECCR) of the First Affiliated Hospital of Wenzhou Medical University.

### Measures

#### Questionnaire design

The survey was cross-sectional and self-administered. The questionnaire was designed by reviewing guidelines and policy documents [22] and journal papers [23, 24] on three dimensions (Knowledge, action, and belief). The questionnaire consisted of four parts: basic information, medical training and skills, engagement in the first line of COVID-19 pandemic prevention and management, and perception of primary doctors' role. The four parts of the questionnaire were detailed as follows: ① Demographic information, including gender (male, female), age (< 40 years old, ≥ 40 years old), education (junior college student and below, undergraduate and above), years of working (≤ 10 years, 10–20 years, > 20 years), professional title (primary professional title and below, middle or senior professional title), and workplace (community health service station, community health service center or primary hospital). ② Medical training and skills, including having received general practice standardized residency training or transfer training (yes or no) and knowing a safe diagnostic strategy (yes or no). ③ Engagement in the first line of COVID-19 pandemic prevention and management activities, including reading authoritative COVID-19 guidelines (yes or no) and participating in the prevention of this epidemic (yes or no). ④ Role perception, including reporting and referring suspected cases, diagnosing and classifying COVID-19, treating suspected cases, and following up with treated patients. Detailed information of the questionnaire is presented in the supplementary file S1.

#### Data-collection procedures

"Wenjuanxing" is a professional online questionnaire survey platform, which provides a WYSIWYG design questionnaire interface. Questionnaire links can be sent to interviewees through WeChat, QQ, Weibo, email and other social platforms. After the survey is completed, the statistical charts can be downloaded to a Word file for online SPSS analysis or original data can be downloaded to Excel to import SPSS software for further analysis. It has the advantages of high efficiency, high quality and low cost. In this study, Wenjuanxing was used to design the questionnaire, which is distributed and collected through the WeChat social platform. Participants completed the questionnaire anonymously and voluntarily. Once the participants concluded the questionnaire, the data were retrieved from Wenjuanxing.

### Definitions

Inclusion and exclusion criteria: The participants in this study were primary care doctors who work in community health service stations, community health service centers, or community hospitals excluding those working in secondary and tertiary hospitals and the staff of support sections and allied health professionals of hospitals.

Engagement in the first line of COVID-19 pandemic prevention and management: primary care doctors reported if they may have been exposed to COVID-19 during the prevention process (e.g., temperature monitoring, suspected cases identification, and triaging in fever clinics).

Reading authoritative COVID-19 guide: Participants reported if they read any version of the COVID-19 guide issued by the authority.

Role perception: the primary care doctors' role positioning was selected by reading the authoritative COVID-19 guide (http://www.nhc.gov.cn), recommendations for preventing and controlling COVID-19 in primary care [25]. Experts suggest that primary care doctors should be educated on infectious diseases, report and refer suspected cases, and follow-up with the treated patients, but should not diagnose and classify COVID-19 or treat suspected cases.

### Statistical analysis

The participant demographics and responses to the questionnaire were summarized with descriptive statistics. Chi-squared tests were used to analyze the factors associated with the role perceptions of primary care doctors. Multivariate analysis was conducted using binary logistic regression to assess further the independent factors related to the consistency of the role perception of primary care doctors with expert advice. All statistical analyses were carried out using SPSS software version 20.0 (SPSS, Inc., Chicago). A p-value of < 0.05 was considered to be significant.

## Results

### Basic information

**Fig. 1:**
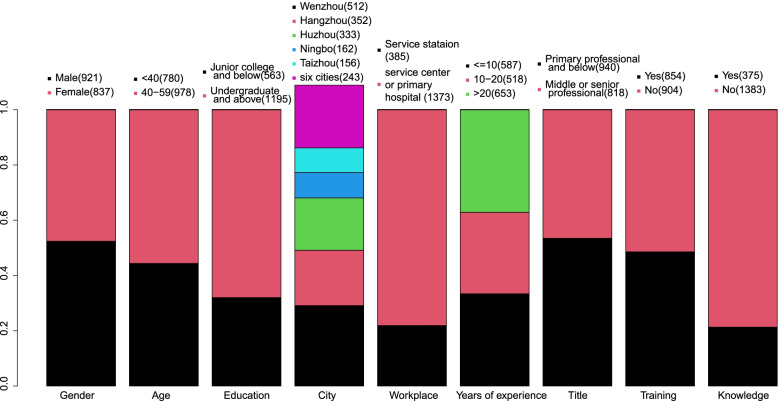
Summary of participants' demographic information and educational background Title, training, and knowledge mean professional title, general practice standardized residency training or job-transfer training, and a safe diagnostic strategy and professional general practice knowledge

In total, 1,758 primary care doctors filled out the questionnaire. There were more males (*n* = 921) than females (*n* = 837), and more than half were aged 40 years and over (*n* = 978). Besides, 68.0% were undergraduate and above (*n* = 1,195). Nearly 80% of participants worked in community health or community hospitals (*n* = 1,373). In comparison, less than 20% of them (*n* = 385) worked in community health stations, which are much smaller and simpler units included in, or affiliated with, community health centers or hospitals. There is an evident variation in their clinical experience exploration and their position in the professional hierarchy. For instance, more than a third of them have over 20 years of clinical experience (*n* = 653), while another third has less than ten years of clinical practice (*n* = 587). More than half of them are positioned on the lowest level of professional entitlement (*n* = 940) (Fig. 1).

Less than half of the participants (*n* = 854) have received general practice standardized residency training or job-transfer training. Therefore, their abilities depend on their hospital's level and their individual endeavor. Besides, only 21.3% (*n* = 375) know a safe diagnostic strategy and show general professional practice knowledge (Fig. 1).

### Perception of the role

**Fig. 2:**
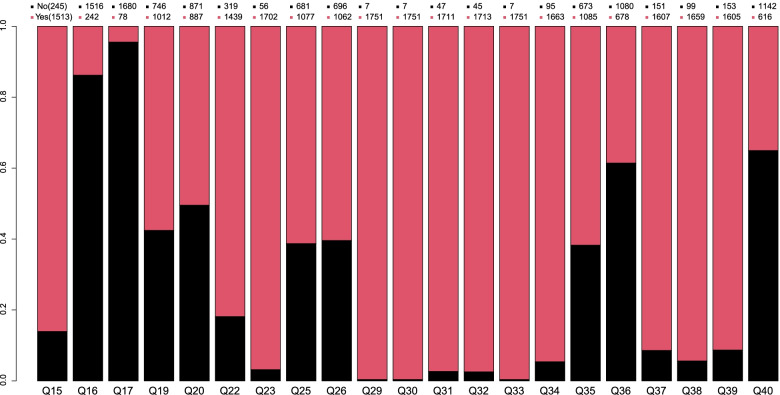
Result distribution of responses to 21 critical questions selected from the questionnaire. Q15: Have you participated in the anti-epidemic work? Q16: Have you encountered patients suspected or confirmed to be infected with the COVID-19 during the anti-epidemic period? Q17: Have you given early medication to patients with suspected or confirmed COVID-19 infection? Q19: Do you think you have the ability to master the diagnostic criteria and classification of novel coronavirus pneumonia? Q20: Do you think the primary doctor should make a diagnosis or classification? Q22: Do you think you have the ability to master the reporting and referral requirements for suspected cases? Q23: Do you think primary doctors should report or refer? Q25: Do you think you have the ability to master the entry and release criteria for the isolation of COVID-19? Q26: Do you think primary-level doctors should take action for the isolation of patients or announce the release of isolation? Q29: Do you think primary doctors should take health education and health promotion on infectious diseases? Q30: Do you think primary doctors should guide the community to accept and use correct information? Q31: Do you think primary doctors should clarify or correct rumors? Q32: Do you think primary doctors should offer public psychological counseling? Q33: Do you think primary doctors should inform community residents when to see a doctor or report a condition in a timely manner? Q34: Do you think primary doctors should identify suspected cases (e.g., by collecting epidemiological history)? Q35: Do you think primary doctors should diagnose suspected cases? Q36: Do you think primary doctors should treat suspected patients (Chinese and Western medicines, alternative methods, non-drug measures)? Q37: Do you think primary doctors should report suspected cases? Q38: Do you think primary doctors should participate in community governance (such as inspection and isolation)? Q39: Do you think primary doctors should follow up of treated patients (such as isolation, medication, and health monitoring)?

To investigate the consistency between primary care doctors' perception of their role and expert advice, we set up 26 questions, including whether suspected or confirmed COVID-19 infected patients had been encountered and whether health education should be related to infectious diseases and health promotion. The results of a survey of 1,758 primary care doctors on these issues is shown in Fig. 2. The majority of the enrolled doctors engaged in the first line of COVID-19 pandemic prevention and management. Of 1,758 primary care doctors, 98.7% reported reading the authoritative COVID-19 guide, and 86.1% engaged in prevention and management. In addition, most primary care doctors agreed that they should carry out health education on infectious diseases, report and refer suspected cases, and follow up with the treated patients (Fig. 2). These three perceptions of the role are highly consistent with expert advice. In contrast, the primary care doctors' perceptions of their role are somewhat inconsistent with expert advice. For instance, 887 (50.5%) thought that primary care doctors could or should diagnose and classify COVID-19, and 1,080 (61.4%) thought primary care doctors could not or should not treat suspected cases. These two perceptions of role had low consistency with expert advice.

### The analysis of low consistency between primary care doctors' perceptions of role and expert advice

#### Comparison of variables among different choices over role positioning of diagnosing and classifying COVID-19

The factors associated with the consistency of primary care doctors' role perception in diagnosing and classifying COVID-19 are described in Table 1. A significant association was observed between primary care doctors' role perception of diagnosing and classifying COVID-19 and workplace (OR 0.635; 95%CI 0.502–0.804), professional title (OR 1.294; 95%CI 1.051–1.592), and also with knowing a safe diagnostic strategy (OR 1.443; 95%CI 1.143–1.822) (all *p* < 0.05). The statistical results of the association of primary care doctors' role perception in diagnosing and classifying COVID-19 and variables are shown in Table S1.

**Table 1:** Logistic regression analysis of the factors associated with consistency of role perception of diagnosis and classification with expert advice from primary care doctors

Variable		OR (95% CI)	*P*
Age			
	≥ 40 years	0.893 (0.646–1.234)	0.492
Workplace			
	Community health service center or primary hospital	0.635 (0.502–0.804)	< 0.001^***^
Years of working			0.154
	10–20 years	1.335 (0.983–1.813)	0.064
	> 20 years	1.412 (0.957–2.085)	0.082
Professional title			
	Middle or senior professional title	1.294 (1.051–1.592)	0.015 ^*^
Knowing a safe diagnostic strategy			
	Yes	0.693 (0.549–0.875)	0.002^**^
Participating in this epidemic prevention			
	Yes	1.303 (0.990–1.722)	0.059

Note: * represent significant *p*-value (* *p* < 0.05, ** *p* < 0.01, *** *p* < 0.001)

#### Comparison of variables among different choices over role perception of mastering the requirements for reporting and referral of suspected cases

Factors associated with consistency of primary care doctors' role perception of mastering the requirements for reporting and referral of suspected cases are described in Table 2. The primary care doctors' role perception of mastering the requirements for reporting and referral of suspected cases was significantly correlated with gender (OR 0.685; 95% CI 0.531–0.883), education (OR 1.830; 95% CI 1.397–2.396), training (OR 1.386; 95% CI 1.074–1.790), knowledge of safe diagnostic strategies (OR 2.641; 95% CI 1.803–3.869), and participation in the prevention of the epidemic (OR 1.437; 95% CI 1.031–2.003). The most significant factor is education and knowledge of safe diagnostic strategies. Among the primary care doctors who gave answers inconsistent with the expert's advice, 24.1% had an education level equal to or below that of the junior college student, and 57.8% had undergraduate degrees or higher levels of education. In contrast, only 10.2% of primary care doctors with a bachelor's degree or above held role perceptions consistent with expert advice (Table S2).

**Table 2:** Logistic regression analysis of the factors associated with consistency of role perception of mastering the requirements for reporting and referral of suspected cases with expert advice in primary care doctors

Variable		OR (95% CI)	*P*
Gender			
	Male	0.685 (0.531–0.883)	0.004^**^
Education			
	Undergraduate and above	1.830 (1.397–2.396)	< 0.001^***^
Professional title			
	Middle or senior professional title	1.287 (0.997–1.674)	0.060
Training			
Yes		1.386 (1.074–1.790)	0.012^*^
Knowing a safe diagnostic strategy			
	Yes	2.641 (1.803–3.869)	< 0.001^***^
Reading authoritative COVID-19 guide			
Yes		2.097 (0.870–5.056)	0.099
Participating in this epidemic prevention			
	Yes	1.437 (1.031–2.003)	0.032^*^

Note: * represent significant *p*-value (* *p *< 0.05, ** *p* < 0.01, *** p < 0.001)

#### Comparison of variables among different choices over role perception of treating suspected cases

The factors associated with the consistency of primary care doctors' role perception in treating suspected cases are described in Table 3. A significant association was observed between primary care doctors' role perception in treating suspected patients and the workplace (OR 0.721; 95%CI 0.565–0.920), professional title (OR 1.449; 95%CI 1.188–1.767), knowing a safe diagnostic strategy (OR 0.709; 95%CI 0.561–0.895), and participating in this epidemic prevention (OR 1.664; 95%CI 1.265–2.188) (all *p* < 0.05). The statistical results of primary care doctors' role perception in treating suspected cases are shown in Table S3. The results showed that among the primary health care doctors who were inconsistent with the expert's advice, 549 worked in community health service centers or primary hospitals, accounting for 81%, and only 19% worked in community health service stations.

**Table 3:** Logistic regression analysis of the factors associated with consistency of role perception of treating suspected cases with expert advice from primary care doctors

Variable		OR (95% CI)	*P*
Workplace			
	Community health service center or primary hospital	0.721 (0.565–0.920)	0.009^**^
Professional title			
	Middle or senior professional title	1.449 (1.188–1.767)	< 0.001***
Knowing a safe diagnostic strategy			
	Yes	0.709 (0.561–0.895)	0.004^**^
Participating in this epidemic prevention			
	Yes	1.664 (1.265–2.188)	< 0.001^***^

Note: * represent significant *p*-value (* p < 0.05, ** *p* < 0.01, *** *p* < 0.001)

#### Comparison of variables among different choices over role perception of following up the treated COVID-19 patients

The factors associated with the consistency of primary care doctors' role perception of following up the treated COVID-19 patients are described in Table 4. A significant association was observed between primary care doctors' perception of treating suspected cases and the workplace (OR 1.612; 95%CI 1.113–2.337, *p* = 0.012) and participating in this epidemic prevention (OR 1.721; 95%CI 1.118–2.649, *P* = 0.014). The statistical results of the association of primary care doctors' role perception of treating suspected cases and variables are shown in Table S4.

**Table 4:** Logistic regression analysis of the factors associated with consistency of role perception of following up the treated COVID-19 patients with expert advice from primary care doctors

Variable		OR (95% CI)	*P*
Age (take < 40 years as the reference)			
	≥ 40 years	0.612 (0.345–1.088)	0.094
Workplace			
	Community health service center or primary hospital	1.612 (1.113–2.337)	0.012^*^
Years of working (take ≤ 10 years as the reference)			0.249
	10–20 years	0.7 (0.348–1.408)	0.317
	> 20 years	1.135 (0.746–1.729)	0.554
Participating in this epidemic prevention			
	Yes	1.721 (1.118–2.649)	0.014^*^

Note: * represent significant *p*-value (* *p* < 0.05, ** *p* < 0.01, *** *p* < 0.001)

## Discussion

Since the first case of unexplained pneumonia was reported in December 2019, the COVID-19 epidemic has widely spread because of its high contagiousness [26, 27]. Countries like the United States, the United Kingdom, and South Korea have published guidelines for controlling the epidemic [28–32]. In the ongoing COVID-19 pandemic, identifying high-risk individuals can address three problems: the underestimation of the actual death risk, the substantial number of asymptomatic and mildly infected individuals, and diagnostic laboratory test errors [33, 34]. China has taken strong measures, including quarantine and population movement restrictions, to prevent the further spread of the epidemic and cut off its transmission [35]. Communities are critical regions in epidemic prevention and control, requiring inch-by-inch management to ensure the implementation of prevention and control measures. The primary medical institutions cooperated with the functional departments of the community to guide the public to take personal protective measures and promptly see a doctor when symptoms appear by disseminating information on epidemic prevention and control [36].

In fighting the COVID-19 pandemic, primary care doctors, 86.1% of whom are working on the front lines, are critical for preventing and managing disease and are a vital part of the Chinese State Council's Joint Prevention and Control mechanism. The Chinese Disease Control and Prevention Center has issued community-based epidemic prevention and control guidelines [22]. These guidelines instruct primary care doctors to participate in reporting and referring suspected cases, epidemiological investigation, specimen collection, management of hospital-acquired infection, personal protection, and improving prevention and control capacity at the community level. Some experts believe that primary care doctors should immediately refer suspected cases to specialized hospitals for diagnosis and treatment once the patient is suspected because of a lack of medical equipment and expertise in primary care [37].

This study showed that over 90% of primary care doctors have a role perception consistent with expert advice regarding health education on infectious diseases, reporting and referral of suspected cases, and following up with treated patients. This indicates that primary care doctors have enough consciousness to carry out the abovementioned duties. Meanwhile, in terms of health education for primary prevention and follow-up of patients in tertiary prevention, primary care doctors have a high consistency with the guidelines and can understand their responsibilities at work. Most primary health care doctors (86.2%) believed that health education on infectious diseases should be carried out. Information targeting the public is the preferred method for controlling the epidemic, and health education remains necessary to increase Knowledge about COVID-19 [38]. Some studies showed that the US, the UK, and Japanese citizens' protection against COVID-19 was lower [39, 40] than that of Chinese citizens [41], which might be related to the excellent information dissemination in China. A timely referral is one of the ways to control the epidemic by blocking transmission. In addition, there is a relatively high incidence of positive viral nucleic acid in patients who met the discharge criteria [42]. If primary care doctors implement the role positioning of follow-up dutifully, the spread of COVID-19 could be slowed.

However, the doctor's perception of their role in the diagnosis and classification of COVID-19 and in treating suspected cases has a low consistency with expert advice, demonstrating a polarizing trend. The proportions of doctors who believe they "should" and "should not" do the above are approximately equal. These gaps may be reflected in the attitude of doctors. Most primary care doctors consider their work less valuable because most patients in the community usually seek treatment with fever, cough, diarrhea and other symptoms, but these symptoms are similar to COVID-19, once too many patients are referred to the higher-level hospital, the workload of primary care doctors will be reduced, and the realization of their self-worth may be weakened [43]. This is the reason why some primary care doctors think that they should diagnose and treat suspected COVID-19 cases, but in infectious diseases it is very dangerous and inappropriate to treat suspected COVID-19 cases without proper protection. The role perception of doctors reflects the knowledge and skills (internal and necessary demand) of clinical problems and the external factors of their diagnosis and treatment supporting (external and sufficient demand). Moreover, there is a trustee of balance between self-cognition and actual competency. Trust between organizations and workers is an essential element of the willingness of professionals to work during a public health crisis, which encourages social interactions and cooperation among health professionals and helps improve care [44]. We suggest implementing training and guidance to enhance the knowledge and ability to diagnose and classify COVID-19 and treat suspected cases, and improving external and sufficient demand like strengthening regional teamwork and supporting primary care doctors to perform competently in epidemic prevention teams.

A study of medical consortia showed that primary care doctors with a senior professional title are more familiar with medical consortia than those with a primary or lower professional title [45]. Our work indicates a significant positive association between the consistency of role perception with expert advice and middle or senior professional title, which is consistent with previous research. Therefore, we consider primary care doctors of middle or senior professional titles as critical primary care providers. They have a clear perception of their role and know if their primary care sites are provided with conditions of diagnosis and treatment or not, while junior doctors lack of such a perception. Due to the late start of China's general practitioners' team construction, there is still an imbalance in terms of professional titles among the general practitioner team [46]. Therefore, primary care doctors with a middle or senior professional title should lead epidemic prevention. A high-quality structural echelon should be formed with doctors having a senior professional title as the backbone and a technical instructor to fully use their superiority in experience, knowledge, and understanding [47].

This paper also found that the workplace can impact the consistency between role perception and the experts' advice. For example, primary care doctors that work in community health service stations have a higher consistency with expert advice than those working in the community health service center or primary hospital. They worked at poor medical conditions of the health service center, but they have strong sense of responsibility and abilities of referral, and routine referrals are also understood by patients. A possible explanation is that primary-level hospitals provide fundamental medical services in relatively smaller areas where staff tend to know their patients better and tend to develop better patient-physician relationships, making them less likely to suffer from violence. Compared to working in community health service stations, doctors working in the community health service center or primary hospital have more stress. Not only strict quarantine policies can lead to verbal violence among patients who visit health centers, and low-income, but high-risk work make they did not deepen the awareness of epidemic referral. One study suggested that mistreatment was common among physicians was associated with occupational distress [48], although another evidence showed that the trust between patient and medical workers in China increased during the pandemic [49]. When a new epidemic or a post-pandemic era comes, we still need to pay attention to primary-level hospitals, because death, work expectation and personal will also increase the pressure on primary health care doctors [50]. Therefore, we should strengthen the training of primary care doctors in community health service centers and primary hospitals.

Surprisingly, knowing a safe diagnostic strategy negatively influences the consistency of role perception with expert advice. Murtagh's general practice [51] records a secure diagnostic method for diagnosis and treatment, guiding primary care doctors in their work. However, when infectious diseases emerge, doctors who understand the safe diagnostic strategy may ignore the specific illness and incorrectly understand their roles. Most primary care doctors (61.7%) could diagnose suspected cases. Community health service centers or primary hospitals are the most grass-roots units in preventing and controlling infectious diseases. Still, Chinese primary care doctors lack experience and abilities in diagnosing and treating infectious diseases in their daily work, which is a problem because the management of infectious diseases is not standardized [52, 53]. In terms of preventing the spread of infections or improving the cure rate of patients, doctors should refer patients to specialized hospitals with isolation conditions and a high level of diagnosis and treatment in a timely fashion. Hence, it is vital to issue authoritative guides for primary care doctors to correct their role in a significant public health emergency.

Under an epidemic, the whole country, including its medical and healthcare system and medical staff, should learn epidemic prevention knowledge and perform their duties based on an accurate perception of their roles [54]. Primary care doctors should define their position based on a thorough consideration of their competence and supporting conditions. This can only be achieved through constant learning, but it ultimately depends on notional responsibilities and personal confidence. The role perception is related to the availability and consensus of epidemiological characteristics, the path of disease development, and the diagnostic criteria and treatment. Even if they tend not to treat suspected cases, we found that engagement in the first line of COVID-19 pandemic prevention and management can help primary doctors with dynamic self-learning and their adjustment to duties. However, most primary care doctors have an unclear perception of their role, which results in their frustration with the lack of a clear central direction or a clinical care model from expert guidelines [55]. Therefore, refining guidelines to confirm the clear role perception of primary care doctors is necessary.

## Conclusions

Most primary care doctors from Zhejiang, China, have participated in COVID-19 pandemic prevention. Nevertheless, their perception of their role in over-diagnosing and classifying COVID-19 and treating suspected cases is inconsistent with expert advice. Therefore, it is vital to guide primary care doctors especially for those with low profile title develop a clear perception of their duties in diagnosis and treatment.

## Supplementary Information

**Additional file 1. **Questionnaire of role perception of primary care doctors under the epidemic situation of COVID-19.

**Additional file 2: Table S1.** Chi-square analysis of the factors associated with consistency of role perception of diagnosis and classification with expert advice in primary care doctors.

**Additional file 3: Table S2.** Chi-square analysis of the factors associated with consistency of role perception of mastering the requirements for reporting and referral of suspected cases with expert advice in primary care doctors.

**Additional file 4:** **Table S3.** Chi-square analysis of the factors associated with consistency of role perception of treating suspected cases with expert advice in primary care doctors.

**Additional file 5: Table S4.** Chi-square analysis of the factors associated with consistency of role perception of following up the treated COVID-19 patients with expert advice in primary care doctors.

## Data Availability

The datasets used and/or analysed during the current study are available from the corresponding author on reasonable request.

## Abbreviations

COVID-19: Coronavirus Disease 2019
OR: Odds ratio
