# Supplementary material for: The roles of primary care doctors in the COVID-19 pandemic: consistency and influencing factors of doctor's perception and actions and nominal definitions
Source: BMC Health Serv Res. 2022 Sep 9;22:1143. doi: 10.1186/s12913-022-08487-0 (PMC9462892; doi:10.1186/s12913-022-08487-0)
Supplement: Supplementary file 1 — Additional file 1. Questionnaire of role perception of primary care doctors under the epidemic situation of COVID-19. [file 12913_2022_8487_MOESM1_ESM.docx]

**Questionnaire of role perception of primary care doctors under the epidemic situation of COVID-19**

**Serial No**. :

**IP address**: 39.186.160.105 (Zhejiang Huzhou)

**Source channel**: Wechat

**Filling time:**

**Filling instructions:** to ensure the accuracy of the survey results, please answer all questions truthfully. Please tick "√" on the questionnaire options or fill in the text answers in the blank.

**Your basic information**

1. Gender: *

□Female □Male

2. Age (years)*

3. Working years (years)*

4. Where you work (City)*

5. Which hospital do you currently work for *

□Village clinic or health station

□Community Health Centre

□County Hospital

□Others

6. What is your current position? *

□Clinician

□Nurse

□Pharmacist

□Public health personnel

□Others

7. What is your educational background?*

□Technical secondary school and below

□Junior college

□Undergraduate

□Master degree or above

8. What is your professional title? *

□Junior professional title and below

□Intermediate title

□Senior title

9. What general practice/basic medical and health service training have you attended? *

□Standardized resident training and job-transfer training

□Have not participated in standardized resident training and job-transfer training

10. Do you use BATHE consultation mode diagnosis when contacting patients?*

□Understand

□Do not understand

□Master the BATHE consultation mode diagnosis, but have no practical application

□Master the BATHE consultation mode diagnosis and have practical applications

11. Do you use RICE consultation mode diagnosis when contacting patients?*

□Understand

□Do not understand

□Master the RICE consultation mode diagnosis, but have no practical application

□Master the RICE consultation mode diagnosis and have practical applications

12. Do you usually use the 5-step method of Motta safety diagnosis in clinical diagnosis?*

□Understand

□Do not understand

□Master the 5-step method of Motta safety diagnosis, but have no practical application

□Master the 5-step method of Motta safety diagnosis and have practical applications

**During this prevention and control COVID-19 epidemic**

13. Have you read any version of the guidelines for COVID-19 issued by the National Health Commission?*

□Read the full text

□Partial reading, or listening to or reading part of the introduction

□Didn't follow the guide; just read some popular science materials

□No attention at all

14. How did you learn about the diagnosis and treatment of new coronary pneumonia? (multiple choice) *

□Official only

□Unofficial only

□ Both official and unofficial

15. What work have you participated in during this COVID-19 epidemic period (multiple choice) *

□Self-quarantine

□Work at the original position

□Fever clinic

□Centralized isolation point medical work

□Temperature detection and palpation at card point

□Patient transport

□Publicity to residents

□Others

16. Have you encountered any suspected or confirmed SARS-CoV-2 infected patients during the COVID-19 epidemic period?*

□Yes

□No

17. Have you taken any early medication for patients suspected or diagnosed with novel coronavirus infection?*

□Yes

□No

18. What is your registration method after checking patients?*

□No participation in checking

□Using paper forms

□Using spreadsheets

19. Do you think you have the ability to master the diagnostic criteria and classification of new coronary pneumonia?*

□Yes

□No

20. Do you think primary doctors should make diagnoses or classifications?*

□Yes

□No

21. If not, which institution or doctor do you think should make the COVID-19 diagnosis?

22. Do you think you have the ability to master the reporting and referral requirements for suspected cases?*

□Yes

□No

23. Do you think primary doctors should report or refer suspected COVID-19 cases?*

□Yes

□No

24. If not, which institution or doctor do you think should report or refer suspected COVID-19 cases?

25. Do you think you have the ability to master the entry and release criteria for isolation of new coronary pneumonia?*

□Yes

□No

26. Do you think primary care doctors should arrange to isolate patients or announce the release of isolation?*

□Yes

□No

27. If not, which institution or doctor do you think should arrange or remove the isolation?*

28. What do you think is the most deficient protective material for the grassroots in the fight against epidemic diseases (please select the most deficient one)?*

Mask

□Protective eyewear

□Glove

□Protective clothing

□Sterilized articles

□Others

**What specific tasks do you think primary doctors should undertake under this COVID-19 pandemic?**

29. Should primary care doctors undertake the task of the health education and health promotion of infectious diseases*

□Yes

□No

30. Should primary care doctors guide the community to accept and use correct information?*

□Yes

□No

31. Should primary care doctors clarify or correct rumors?*

□Yes

□No

32. Should primary care doctors undertake public psychological counseling and mental health counseling?*

□Yes

□No

33. Should primary care doctors undertake to inform the community residents under what circumstances to see a doctor or report their illness in time?*

□Yes

□No

34. Should primary care doctors investigate suspected cases (by collecting epidemiological history, etc.)*

□Yes

□No

35. Should primary care doctors diagnose suspected cases*

□Yes

□No

36. Should primary care doctors treatment of suspected cases (Chinese and Western medicines, alternative methods, non-pharmaceutical measures)?*

□Yes

□No

37. Should primary care doctors report suspected cases*

□Yes

□No

38. Should primary care doctors participate in community governance (patrol, isolation, etc.)?*

□Yes

□No

39. Should primary care doctors follow up on the treated patients (isolation, medication, health monitoring, etc.)?*

□Yes

□No

40. What specific tasks should other grassroots medical personnel undertake
