# Supplementary material for: The roles of primary care doctors in the COVID-19 pandemic: consistency and influencing factors of doctor's perception and actions and nominal definitions
Source: BMC Health Serv Res. 2022 Sep 9;22:1143. doi: 10.1186/s12913-022-08487-0 (PMC9462892; doi:10.1186/s12913-022-08487-0)
Supplement: Supplementary file 2 — Additional file 2: Table S1. Chi-square analysis of the factors associated with consistency of role perception of diagnosis and classification with expert advice in primary care doctors. [file 12913_2022_8487_MOESM2_ESM.docx]

Table S1. Chi-square analysis of the factors associated with consistency of role perception of diagnosis and classification with expert advice in primary care doctors

|  |  | diagnosing and classifying COVID-19 | |
| --- | --- | --- | --- |
|  |  | Inconsistent | Consistent |
| sex | |  |  |
|  | male | 461(52.0) | 460(52.8) |
|  | female | 426(48.0) | 411(47.2) |
|  | *P*(χ^2^) | 0.724(0.124) | |
| age | |  |  |
|  | <40 | 417(47.0) | 363(41.7) |
|  | ≥40 | 470(53.0) | 508(58.3) |
|  | *P*(χ^2^) | 0.024(5.070) | |
| education | |  |  |
|  | junior college student and below | 269(30.3) | 294(33.8) |
|  | undergraduate and above | 618(69.7) | 577(66.2) |
|  | *P*(χ^2^) | 0.124(2.371) | |
| workplace | |  |  |
|  | community health service  station | 163(18.4) | 222(25.5) |
|  | community health service  center or primary hospital | 724(81.6) | 649(74.5) |
|  | *P*(χ^2^) | <0.001(12.994) | |
| years of experience | | |  |
|  | ≤10 | 330(37.2) | 257(29.5) |
|  | 10-20 | 248(28.0) | 270(31.0) |
|  | >20 | 309(34.8) | 344(39.5) |
|  | *P*(χ^2^) | 0.003(11.744) | |
| professional title | | |  |
|  | primary professional title and below | 506(57.0) | 434(49.8) |
|  | middle or senior professional title | 381(43.0) | 437(50.2) |
|  | *P*(χ^2^) | 0.002(9.204) | |
| training^a^ | | |  |
|  | yes | 450(50.7) | 404(46.4) |
|  | no | 437(49.3) | 467(53.6) |
|  | *P*(χ^2^) | 0.068(3.328) | |
| knowing a safe diagnostic strategy | | |  |
|  | yes | 217(24.5) | 158(18.1) |
|  | no | 670(75.5) | 713(81.9) |
|  | *P*(χ^2^) | 0.001(10.475) | |
| reading authoritative COVID-19 guide | | |  |
|  | yes | 874(98.5) | 862(99.0) |
|  | no | 13(1.5) | 9(1.0) |
|  | *P*(χ^2^) | 0.415(0.665) | |
| participating in this epidemic prevention | | |  |
|  | yes | 746(84.1) | 767(88.1) |
|  | no | 141(15.9) | 104(11.9) |
|  | *P*(χ^2^) | 0.017(5.734) | |

a: received general practice standardized residency training or job-transfer training
