# Supplementary material for: The roles of primary care doctors in the COVID-19 pandemic: consistency and influencing factors of doctor's perception and actions and nominal definitions
Source: BMC Health Serv Res. 2022 Sep 9;22:1143. doi: 10.1186/s12913-022-08487-0 (PMC9462892; doi:10.1186/s12913-022-08487-0)
Supplement: Supplementary file 3 — Additional file 3: Table S2. Chi-square analysis of the factors associated with consistency of role perception of mastering the requirements for reporting and referral of suspected cases with expert advice in primary care doctors. [file 12913_2022_8487_MOESM3_ESM.docx]

**Table S2** Chi-square analysis of the factors associated with consistency of role perception of mastering the requirements for reporting and referral of suspected cases with expert advice in primary care doctors

|  |  | Report and master suspected cases | |
| --- | --- | --- | --- |
|  |  | Inconsistent | Consistent |
| sex | |  |  |
|  | male | 775(44.1) | 146(8.3) |
|  | female | 664(37.8) | 173(9.8) |
|  | *P*(χ^2^) | 0.009(6.849) | |
| age | |  |  |
|  | <40 | 653(37.1) | 127(7.2) |
|  | ≥40 | 786(44.7) | 192(10.9) |
|  | *P*(χ^2^) | 0.070(3.278) | |
| education | |  |  |
|  | junior college student and below | 423(24.1) | 140(8.0) |
|  | undergraduate and above | 1016(57.8) | 179(10.2) |
|  | *P*(χ^2^) | <0.001(25.190) | |
| workplace | |  |  |
|  | community health service  station | 303(17.2) | 82(4.7) |
|  | community health service  center or primary hospital | 1136(64.6) | 237(13.5) |
|  | *P*(χ^2^) | 0.069(3.300) | |
| years of experience | | |  |
|  | ≤10 | 490(27.9) | 97(5.5) |
|  | 10-20 | 417(23.7) | 101(5.7) |
|  | ＞20 | 532(30.3) | 121(6.9) |
|  | *P*(χ^2^) | 0.419(1.741) | |
| professional title | | |  |
|  | primary professional title and below | 743(42.3) | 197(11.2) |
|  | middle or senior professional title | 696(39.6) | 122(6.9) |
|  | *P*(χ^2^) | 0.001(10.754) | |
| training^a^ | | |  |
|  | yes | 729(41.5) | 125(7.1) |
|  | no | 710(40.4) | 194(11.0) |
|  | *P*(χ^2^) | <0.001(13.765) | |
| knowing a safe diagnostic strategy | | |  |
|  | yes | 341(19.4) | 34(1.9) |
|  | no | 1098(62.5) | 285(16.2) |
|  | *P*(χ^2^) | <0.001(26.454) | |
| reading authoritative COVID-19 guide | | |  |
|  | yes | 1426(81.1) | 310(17.6) |
|  | no | 13(0.7) | 9(0.5) |
|  | *P*(χ^2^) | 0.005(7.772) | |
| participating in this epidemic prevention | | |  |
|  | yes | 1252(71.2) | 261(14.8) |
|  | no | 187(10.6) | 58(3.3) |
|  | *P*(χ^2^) | 0.016(5.857) | |

a: received general practice standardized residency training or job-transfer training
