# Supplementary material for: The roles of primary care doctors in the COVID-19 pandemic: consistency and influencing factors of doctor's perception and actions and nominal definitions
Source: BMC Health Serv Res. 2022 Sep 9;22:1143. doi: 10.1186/s12913-022-08487-0 (PMC9462892; doi:10.1186/s12913-022-08487-0)
Supplement: Supplementary file 4 — Additional file 4: Table S3. Chi-square analysis of the factors associated with consistency of role perception of treating suspected cases with expert advice in primary care doctors. [file 12913_2022_8487_MOESM4_ESM.docx]

**Table S3** Chi-square analysis of the factors associated with consistency of role perception of treating suspected cases with expert advice in primary care doctors

|  |  | treating suspected cases | |
| --- | --- | --- | --- |
|  |  | Inconsistent | Consistent |
| sex | |  |  |
|  | male | 367(54.1) | 554(51.3) |
|  | female | 311(45.9) | 526(48.7) |
|  | *P*(χ^2^) | 0.247(1.341) | |
| age | |  |  |
|  | <40 | 296(43.7) | 484(44.8) |
|  | ≥40 | 382(56.3) | 596(55.2) |
|  | *P*(χ^2^) | 0.635(0.226) | |
| education | |  |  |
|  | junior college student and below | 225(33.2) | 338(31.3) |
|  | undergraduate and above | 453(66.8) | 742(68.7) |
|  | *P*(χ^2^) | 0.409(0.683) | |
| workplace | |  |  |
|  | community health service  station | 129(19.0) | 256(23.7) |
|  | community health service  center or primary hospital | 549(81.0) | 824(76.3) |
|  | *P*(χ^2^) | 0.021(5.327) | |
| years of experience | |  |  |
|  | ≤10 | 228(33.6) | 359(33.2) |
|  | 10-20 | 191(28.2) | 327(30.3) |
|  | >20 | 259(38.2) | 394(36.5) |
|  | *P*(χ^2^) | 0.613(0.978) | |
| professional title | |  |  |
|  | primary professional title and below | 399(58.8) | 541(50.1) |
|  | middle or senior professional title | 279(41.2) | 539(49.9) |
|  | *P*(χ^2^) | 0.001(12.838) | |
| training^a^ | |  |  |
|  | yes | 337(49.7) | 517(47.9) |
|  | no | 341(50.3) | 563(52.1) |
|  | *P*(χ^2^) | 0.454(0.561) | |
| knowing a safe diagnostic strategy | |  |  |
|  | yes | 170(25.1) | 205(19.0) |
|  | no | 508(74.9) | 875(81.0) |
|  | *P*(χ^2^) | 0.002(9.212) | |
| reading authoritative COVID-19 guide | |  |  |
|  | yes | 666(98.2) | 1070(99.1) |
|  | no | 12(1.8) | 10(0.9) |
|  | *P*(χ^2^) | 0.121(2.401) | |
| participating in this epidemic prevention | |  |  |
|  | yes | 555(81.9) | 958(88.7) |
|  | no | 123(18.1) | 122(11.3) |
|  | *P*(χ^2^) | <0.001(16.272) | |

a: received general practice standardized residency training or job-transfer training
