# Supplementary material for: The roles of primary care doctors in the COVID-19 pandemic: consistency and influencing factors of doctor's perception and actions and nominal definitions
Source: BMC Health Serv Res. 2022 Sep 9;22:1143. doi: 10.1186/s12913-022-08487-0 (PMC9462892; doi:10.1186/s12913-022-08487-0)
Supplement: Supplementary file 5 — Additional file 5: Table S4. Chi-square analysis of the factors associated with consistency of role perception of following up the treated COVID-19 patients with expert advice in primary care doctors. [file 12913_2022_8487_MOESM5_ESM.docx]

**Table S4** Chi-square analysis of the factors associated with consistency of role perception of following up the treated COVID-19 patients with expert advice in primary care doctors

|  |  | | Follow up the treated patients (isolation, medication, health monitoring, etc.) | |
| --- | --- | --- | --- | --- |
|  |  | | Inconsistent | Consistent |
| sex | | |  |  |
|  | male | | 843(48.0) | 78(4.4) |
|  | female | | 762(43.3) | 75(4.3) |
|  | *P*(χ^2^) | | 0.715(0.133) | |
| age | | |  |  |
|  | <40 | | 736(41.9) | 44(2.5) |
|  | ≥40 | | 869(49.4) | 109(6.2) |
|  | *P*(χ^2^) | | <0.001(16.545) | |
| education | | |  |  |
|  | junior college student and below | | 507(28.8) | 56(3.2) |
|  | undergraduate and above | | 1098(62.5) | 97(5.5) |
|  | *P*(χ^2^) | | 0.204(1.612) | |
| workplace | | |  |  |
|  | community health service  station | | 339(19.3) | 46(2.6) |
|  | community health service  center or primary hospital | | 1266(72.0) | 107(6.1) |
|  | *P*(χ^2^) | | 0.011(6.533) | |
| Years of expression | |  |  |  |
|  | ≤10 | | 557(31.7) | 30(1.7) |
|  | 10-20 | | 466(26.5) | 52(3.0) |
|  | ＞20 | | 582(33.1) | 71(4.0) |
|  | *P*(χ^2^) | | 0.001(14.566) | |
| professional title | |  |  |  |
|  | primary professional title and below | | 862(49.0) | 78(4.4) |
|  | middle or senior professional title | | 743(42.3) | 75(4.3) |
|  | *P*(χ^2^) | | 0.518(0.417) | |
| training^a^ | |  |  |  |
|  | yes | | 786(44.7) | 68(3.9) |
|  | no | | 819(46.6) | 85(4.8) |
|  | *P*(χ^2^) | | 0.284(1.146) | |
| knowing a safe diagnostic strategy | |  |  |  |
|  | yes | | 350(19.9) | 25(1.4) |
|  | no | | 1255(71.4) | 128(7.3) |
|  | *P*(χ^2^) | | 0.115(2.488) | |
| reading authoritative COVID-19 guide | |  |  |  |
|  | yes | | 1586(90.2) | 150(8.5) |
|  | no | | 19(1.1) | 3(0.2) |
|  | *P*(χ^2^) | | 0.409(0.682) | |
| participating in this epidemic prevention | |  |  |  |
|  | yes | | 1390(79.1) | 123(7.0) |
|  | no | | 215(12.2) | 30(1.7) |
|  | *P*(χ^2^) | | 0.034(4.494) | |

a: received general practice standardized residency training or job-transfer training
